# Distractions, analytical thinking and falling for fake news: A survey of psychological factors

**DOI:** 10.1057/s41599-023-01813-9

**Published:** 2023-06-12

**Authors:** Adrian Kwek, Luke Peh, Josef Tan, Jin Xing Lee

**Affiliations:** 1 College of Interdisciplinary and Experiential Learning, Singapore University of Social Sciences, Singapore, Singapore; 2 School of Science and Technology, Singapore University of Social Sciences, Singapore, Singapore; 3 Curriculum Planning and Development Division, Ministry of Education, Singapore, Singapore; 4 School of Computing, National University of Singapore, Singapore, Singapore

**Keywords:** Cultural and media studies, Cultural and media studies, Science, technology and society, Psychology, Education

## Abstract

Analytical thinking safeguards us against believing or spreading fake news. In various forms, this common assumption has been reported, investigated, or implemented in fake news education programs. Some have associated this assumption with the inverse claim, that distractions from analytical thinking may render us vulnerable to believing or spreading fake news. This paper surveys the research done between 2016 and 2022 on psychological factors influencing one’s susceptibility to believing or spreading fake news, considers which of the psychological factors are plausible distractors to one’s exercise of analytical thinking, and discusses some implications of considering them as distractors to analytical thinking. From these, the paper draws five conclusions: (1) It is not analytical thinking per se, but analytical thinking directed to evaluating the truth that safeguards us from believing or spreading fake news. (2) While psychological factors can distract us from exercising analytical thinking and they can also distract us in exercising analytical thinking. (3) Whether a psychological factor functions as a distractor from analytical thinking or in analytical thinking may depend on contextual factors. (4) Measurements of analytical thinking may not indicate vulnerability to believing or spreading fake news. (5) The relevance of motivated reasoning to our tendency to believe fake news should not yet be dismissed. These findings may be useful to guide future research in the intersection of analytical thinking and susceptibility to believing or spreading fake news.

## Introduction

Fake news has deleterious effects on society. The effects range from destabilizing society by cleaving racial and religious fault lines (Grambo, 2019), to influencing national elections (Alcott and Gentzkow, 2017), to derailing public health policy implementations (Waszak et al., 2018). Since the gradual appearance and entrenchment of the term “fake news” in the popular lexicon in 2016, much research has been done on the psychological and environmental determinants of people’s responses to fake news, as well as the effectiveness of interventions designed to reduce the deleterious effects of fake news on society. This review begins with Pennycook and Rand’s (2019) observation that people fall for fake news because they are distracted from thinking analytically. After elaborating on Pennycook and Rand’s observation and orienting it to the sharing of fake news, we conduct a survey of the relevant literature from 2016 to 2022 about possible psychological distractors that cause us to fall for fake news.

Disinformation is information that is (i) false, (ii) communicated as true, and (iii) intentionally communicated as true in order to influence people’s beliefs or behavior (see Duffy et al., 2020; McGonagle, 2017). Item (iii) distinguishes disinformation as a type of misinformation. Misinformation is information that is false and communicated as true but without the intention for the impression of its truth to influence people’s beliefs or behavior (see Ireton and Posetti, 2018; Wardle and Derakhshan, 2017). False information that is published as news due to sloppy journalism is an example of misinformation. Disinformation includes intentionally false propaganda, intentionally false advertising, and fake news. Fake news is distinguished from other types of disinformation by being designed to mislead people that the content is news, for example, by mimicking the visual format of reputable news websites.

There are at least three senses of the term “falling for fake news”. When one falls for fake news, one believes the disinformation, cannot distinguish it from genuine news, and/or retransmits it. We take Pennycook and Rand’s (2019) observation that people fall for fake news because they are distracted from thinking analytically as our starting point. Being saved from falling for misinformation of any kind by thinking carefully about the information is a common experience that many people have. This experience lends intuitive support to the assumption that we tend to fall for misinformation when we do not think carefully enough about the information.

For many studies, one’s propensity and competency in “thinking carefully” about information is indicated by the completion of analytical thinking scales. Pennycook and Rand (2018, 2019), Nurse et al. (2022), and Pehlivanoglu et al. (2021, 2022) use the cognitive reflection test (CRT). The CRT measures one’s propensity to engage in slow and careful thinking. It presents the subject with a question that admits to a quick but wrong answer and a correct answer that requires slow and careful thinking. Other tests indicating one’s propensity to think carefully about a subject matter include tests of conscientiousness (Buchanan, 2020; Lawson and Kakkar, 2022; Li et al., 2022) and tests of argumentative ability (Lantian et al., 2021).

While there have been literature reviews about psychological factors that render us susceptible to fake news, we have not found any that focus on the relation between the factors and analytical thinking directly. Due to the large number of disinformation-susceptibility studies investigating psychological factors and analytical thinking, an analytical review of such studies can help us to understand the extent to which analytical thinking can “save” us from falling for fake news.

### Research question

What psychological distractions to analytical thinking mediate the belief in and/or retransmission of fake news?

## Methodology

Taking Pennycook and Rand’s (2019) paper as the seed article for our project, we identified key terms from it and its reference list by relevance to our research question, frequency of usage in journal article titles and abstracts, and terms with similar meanings to these terms that also frequently appear in titles and abstracts. We conducted a critical review (Grant et al., 2009) by searching for relevant literature on our university e-journal database and Google Scholar from 2016 onwards with the following 9 search strings:

1. (spread OR disseminat*) AND (“fake news” OR misinform* OR disinform*) AND (belie* OR identif* OR discern* OR assess* OR rat* OR evaluat*)
2. (spread OR disseminat*) AND (“fake news” OR misinform* OR disinform*)
3. (spread OR disseminat*) AND (“fake news” OR misinform* OR disinform*) AND (belie* OR identif* OR discern* OR assess* OR rat* OR evaluat*) AND (reflect* OR think* OR reflex* OR cogni* OR reason* OR motivated OR bullshit* OR profound)
4. (spread OR disseminat*) AND (“fake news” OR misinform* OR disinform*) AND (reflect* OR think* OR reflex* OR cogni* OR reason* OR motivated OR bullshit* OR profound)
5. (spread OR disseminat*) AND (“fake news” OR misinform* OR disinform*) AND (belie* OR identif* OR discern* OR assess* OR rat* OR evaluat*) AND (heuristic* OR familiar* OR source OR credib* OR “confirmation bias”)
6. (spread OR disseminat*) AND (“fake news” OR misinform* OR disinform*) AND (heuristic* OR familiar* OR source OR credib* OR “confirmation bias”)
7. (spread OR disseminat*) AND (“fake news” OR misinform* OR disinform*) AND (signal* OR reputation OR identity OR virtue OR overclaim*) AND (prosocial OR pro-social OR moral* OR outrage* OR punish* OR interesting* OR entertain* OR gossip* OR rumor)
8. (spread OR disseminat*) AND (“fake news” OR misinform* OR disinform*) AND (signal* OR reputation OR identity OR virtue OR overclaim*)
9. (spread OR disseminat*) AND (“fake news” OR misinform* OR disinform*) AND (prosocial OR pro-social OR moral* OR outrage* OR punish* OR interesting* OR entertain* OR gossip* OR rumor)

For repeated search results, we omitted the redundant items. In order to focus on psychological distractors mediating belief and retransmission of disinformation, we further filtered the search results with the following 5 exclusion criteria and respective rationales:

**Table:** 

	Exclusion criterion	Rationale	Excluded
i.	Exclude studies on digital, media, and science literacy and related topics.	Literacies are associated with a wide range of psychological factors and do not serve to identify individual psychological factors.	12 articles
ii.	Exclude network analyses on the spread of information or related topics.	Network analyses give the pattern of information transmission, which is outside the scope of the research question.	36 articles
iii.	Exclude studies on the effects of labeling information as “fake news” or related topics.	The effects of “fake news” labeling are outside the scope of the research question.	3 articles
iv.	Exclude philosophical analyses into the definition of fake news or related topics.	Philosophical analyses concern abstractions and idealizations that are outside the scope of the research question.	14 articles
v.	Exclude studies that focus on the effects of photographs, videos, and other non-textual information, or compare them with the effects of textual information on belief in or spreading of fake news.	The focus of the research question is analytical thinking, which typically concerns propositional content most clearly expressed textually.	6 articles

## Findings

In this section, we summarize the contents of empirical studies that relate to our research questions. By searching Google Scholar and the journal databases that our university library has access to, and by manual filtering based on the above five criteria, we arrived at 87 relevant articles. In this section, we present our curation of articles according to psychological factors that are covered by research in the 2016–2022 time period.

### Emotions

Emotions can influence one’s responses to fake news. Martel et al. (2020) suggest the following influences on finding fake news persuasive. First, certain moods associated with happiness or higher motivation generally correlate with one’s tendency to believe false information and negatively correlate with one’s capacity to tell when one is being deceived; and conversely, that certain moods associated with sadness or lower motivation generally correlate with doubting and disbelief. Second, when one is angry, one is more likely to depend on heuristic cues in deciding whether to believe information and less likely when one is sad. Third, anxiety tends to render one more willing to entertain perspectives contrary to one’s own, while anger tends to decrease the propensity. Maldonado (2019) suggests that emotion is inextricably intertwined with reasoning, and confirmation bias may elicit positive emotions that have a physiological basis in dopamine production. Thus, certain emotions can not only render us more likely to believe in fake news but also make it pleasurable to do so.

In their own research, Martel et al. (2020) found that depending on one’s emotions in processing false news information made one more likely to believe them. Horner et al. (2021) have shown heightened emotions to positively correlate with finding fake news aligning with one’s existing beliefs persuasive and with disseminating disinformation, as well as withholding information incompatible with existing beliefs. The emotions of anxiety and anger have active causal roles. They cause one to fall for fake news in order to act with the information. In contrast, sadness is passive in the sense that it causes one to miss signs that the information is fake news, and to proceed as though it is genuine news.

Weimann-Saks et al., (2022) were unable to detect a mediating role played by emotions between one’s cognition of the information and one’s subsequent actions based on the cognition. The information in question is rumors, which share with fake news the quality of being information that is unverified but communicated as true. This finding does not detract from the general tenor that dependence on emotions renders one more susceptible to believing fake news as the subsequent actions in question pertain to spreading rather than believing the information. It does, however, indicate that one should separately evaluate the role of emotions in the believing of fake news and the role of emotions in the spreading of fake news.

#### Anxiety

The higher the stakes involved and the greater the perceived risk, the greater the anxiety. Anxiety, in turn, causes one to err on the side of treating the information as true and disseminating it as such, even when one is not sure. This phenomenon was widespread in the spreading of Covid-19 disinformation about ways to detect, prevent or treat the disease during the pandemic by well-meaning family and friends for whom the stakes could be the death of a loved one. Oh and Lee (2019) did a relatively early study linking health anxiety, together with health literacy and one’s perception of the significance of the information, to checking on the veracity of the information and spreading it. Laato et al. (2020) showed that one’s perception of danger posed by Covid-19 together with one’s assessment of susceptibility contributed to one’s propensity to share the information without verification. These studies suggest that the anxiety caused by the fear of death can lead to the spreading of fake news. Sun et al. (2020) showed that the elderly are more apt to retransmit unverified information the more they believe the information and the more anxious they are about it. Su (2021) showed that, when anxiety was taken into consideration as a mediator, the positive relationship between social media use and misinformation beliefs increased in significance. Kim and Kim (2020) showed that apparent risk and stigma increase belief in false information about Covid-19. Li et al. (2022) showed that such propensities are compounded by personality traits like extroversion, emotional instability and conscientiousness. Li et al. (2022) showed that people were more persuaded by Covid-19 misinformation and more likely to share it when their perception of risk from Covid-19 deaths increased and they experienced more intense negative emotions. Ahmed (2022) presents a different type of anxiety—the fear of missing out (FOMO). Ahmed shows FOMO positively correlating with the tendency to retransmit deep fakes, which is enhanced by low cognitive ability or increased social media use.

#### Anger

Chuai and Zhao (2022) showed that increased anger associated with a piece of fake news positively correlates with its increased virality and hypothesize that the anger incentivizes the retransmission of the information. Deng and Chau (2021) showed that consumers of online news become skeptical of content when the content is laced with angry expressions. This shows that the locus of anger is significant. If so, then the information should evoke anger rather than contain angry language. Han et al. (2020) showed that anger increased one’s propensity to disseminate disinformation by causing them to find disinformation scientifically persuasive. Bago et al., (2022) showed that anger is correlated with decreasing truth discernment of headlines, and also that truth discernment improved when the subjects actively reduce their emotions. Moral outrage and the anger that it generates have also been shown to make people believe disinformation. Ali et al. (2022a) studied both fear and anger. They found that fear tended to make people who are skeptical of vaccines share false anti-vaccine information while anger tended to make people who are neither skeptical nor endorsing vaccines share false anti-vaccine information.

### Repeated exposure

Repeated exposure to fake news may cause us to believe or retransmit the information without thinking analytically about its truth. Pennycook et al. (2018) found that even one encounter with disinformation can cause subjects to believe that the information is accurate. They hypothesize that this is due to the fluency of subsequent processing after the initial exposure. The effect persists even when the information is flagged as controversial by debunking services. In a different study about how visual images affect people’s accuracy judgments, Smelter and Calvillo (2020) corroborated Pennycook et al.’s findings that repeated exposure increases people’s propensity to judge false information as accurate. Repeated exposure to disinformation can also increase people’s propensity to retransmit false information. Effron and Raj (2020) found that subjects considered it less ethically wrong to retransmit headlines that are labeled false when they are exposed to them up to four times. This is so even when subjects did not believe the headlines. In Nadarevic et al. (2020)’s investigation of the joint effect of several factors on accuracy evaluations, source credibility together with repeated exposure was found to have a robust effect on accuracy evaluations. Swire et al. (2017) found that familiarity caused by repeated exposure contributes to the continuing influence effect, where subjects believe disinformation even after being informed of its correction. They found the elderly to be more susceptible and found that explanations and attention to the facts work to dispel the continued influence effect.

### Altruism

Altruism is the desire to benefit one’s community without seeking recompense for oneself. It has been identified as one of the most important reasons for retransmitting fake news (Balakrishnan et al., 2021; Apuke and Omar, 2021). This can be as wide as the community of human beings or as parochial as one’s in-group. Anxiety can be associated with altruism when one’s desire to benefit others stems from one’s concern about their wellbeing. The studies that are relevant to altruism as a contributing factor to one’s susceptibility to disinformation are of two types. The first type studies the desire to benefit as a factor. Balakrishnan et al. (2021) found among a Malaysian sample that altruism was a significant factor in accounting for the retransmission of disinformation. The second type studies collectivism as a factor. Duffy and Tan (2022) found that, when unsure whether news information is true or false, subjects would share it because sharing contributes in part to the cohesiveness of the group, just as rumor does. Sun et al. (2020) and Weimann-Saks et al. (2022) also found similarities between the retransmission of disinformation and rumors. Comparing Chinese with American collectivism scores, Lin et al. (2022) found that Chinese subjects with increased collectivism scores tended to interpret arbitrarily produced and unclear messages as communicating significant information. They found that even fleeting experiences of collectivism caused subjects to find meaning in such information.

### Identity protection or enhancement

Factors pertaining to the protection or display of one’s identity can contribute to one’s susceptibility to disinformation. Pennycook and Rand (2020) found that people who “overclaim”, that is, who signal that they know more than what they actually do are more susceptible to believing disinformation. Islam et al. (2020) found, among other factors, that self-promotion is positively correlated with the retransmission of information without checking its veracity. Littrell et al. (2021) found that people who indulge in “persuasive bullshitting”, that is, who produce untruths aiming to impress or convince an audience, are more susceptible to believing disinformation. Pereira et al. (2021) studied the effect of subjects’ interest in protecting their identity with respect to political affiliation on their credence and tendency to share news information and found a positive correlation. This study enables us to link confirmation bias or motivated reasoning with identity in susceptibility to disinformation. Druckman et al. (2021) found that subjects belonging to ethnic groups that are in the minority in society, who are fervently religious, or who have robust political allegiances are more likely to hold false beliefs. However, Islam et al. (2020) had earlier found that being religious is associated with a decrease in retransmission of unconfirmed content.

### Confirmation bias

There are numerous studies on the effect of confirmation bias and people’s propensity to fall for fake news. In a study about confirmation bias in finding information about climate change persuasive and effective, Zhou and Shen (2022) established strong positive correlations. Bauer and Clemm von Hohenberg (2021) found that subjects who were supplied with false information that aligned with their beliefs were more likely to trust news information from the same source. Horner et al. (2021), in a study on emotions driving credence in false headlines and their dissemination, found that subjects had a greater tendency to believe information that supports beliefs they already possess. Tandoc et al. (2021) discovered an association between people’s credence in disinformation and political affiliation. The link between political affiliation and falling for fake news was corroborated by Pereira et al. (2021), who narrowed it to confirmation bias specifically, defined as a preference for news that dovetail with their prior stereotypical beliefs. Michael and Breaux (2021) explored the converse relation, between political affiliation and skepticism. They found that there is a tendency for people to be skeptical about news information -- i.e., regarded as “fake news”-- information that is incompatible with their political stances. This finding is corroborated by Baxter et al. (2019) in Scotland, Bozdağ and Koçer (2022) in Turkey, Hameleers and Brosius (2022) in the Netherlands, and Tsang (2021) in Hong Kong. Michael and Sanson (2021) studied confirmation bias as an effect of the joint influence of political affiliation and choice of news sources, which in turn affect people’s ability to distinguish real from fake news. In a similar vein, Traberg and van der Linden (2022) found that political affiliation mediated by perceptions of source credibility influences people to judge information aligned with their political orientations to be more accurate. Doing a comparison between conspiracy mindset and political affiliation, Faragó et al. (2020) found that the effect of political affiliation mediated by source credibility in influencing people to believe “wishful thinking” political disinformation was greater than that of conspiracy mindset. Specifically pertaining to one’s propensity to believe and one’s propensity to share disinformation, Turel and Osatuyi (2021) found that political affiliation is positively correlated to them, mediated by factors like the political affiliation of one’s social network. Pearson and Knobloch-Westerwick (2019) found that there is less confirmation bias in consuming printed vs. online news. Some studies cast doubt on the strong association between confirmation bias and falling for fake news. Baptista et al. (2021), found that participants with conservative views were more likely to believe and spread disinformation, regardless of their political orientation. Corroborating this finding, Calvillo et al. (2020) found that subjects with a conservative political orientation tend to be less accurate in distinguishing real from fake news.

### Source credibility

The perceived authoritativeness pertaining to source credibility has received much attention (see Buchanan and Benson, 2019; Buchanan, 2020; Pehlivanoglu et al., 2021; Folkvord et al., 2022). Sterrett et al. (2019) distinguish between two factors that lead subjects to find social media news credible—the credibility of the individual who retransmits the information and the credibility of the news platform. Nadarevic et al. (2020) investigated the interaction between the perceived credibility of the source with other factors in truth discrimination. Faragó et al. (2020) found that perceived source credibility is an important mediator between political affiliation and belief in disinformation. The type of information sources that one holds to be authoritative can indicate one’s propensity to fall for fake news. Bonafé-Pontes et al. (2021) found for Brazilian participants that those who trusted the credibility of social media news tended to be worse at truth discrimination; and conversely, those who trust the WHO and traditional media (newspapers, radio and television) tended to be better at truth discrimination. However, Tsang (2021) did not find any association between types of sources and the perception that a piece of “news” is false. Furthermore, Hameleers et al. (2022) found that people who tend to identify fake news are those who tend to distrust mainstream news platforms and who tend to engage more with non-mainstream news platforms. Zimmermann and Kohring (2020) bridge the gap between distrust in news and belief in disinformation. Xiao et al. (2021) found that trust in social media news is an important mediator between the consumption of social media news and conspiracy ideation. De Coninck et al. (2021)’s and Melki et al. (2021)’s results corroborate others’ findings as follows: engagement with traditional media correlates negatively with belief in disinformation and conspiracy theories; engagement with political personalities, social media and personal social networks correlates positively with belief in disinformation and conspiracy theories. However, de Connick et al. (2021) also found that engagement with healthcare specialists correlates negatively with belief in conspiracy theories only, but does not reduce credence in disinformation. In a similar vein, Lobato et al. (2020) found low trust in mainstream medicine was among the factors associated with vulnerability to health disinformation. Hopp et al. (2020) found that people who have extreme beliefs, high distrust of mainstream media and high social distrust are also more likely to retransmit fake news. Finally, Laato et al. (2020) found that subjects’ propensity to retransmit information without checking its veracity is associated with their faith in information on the internet coupled with having to cope with too much information.

### Social endorsement

The perception of social endorsement constitutes a second basis for people’s trust in the veracity of a message or willingness to share it. Studies about the perception of social endorsement can be subdivided into those where trust is based on some perceived reputational aspect of the source that is unrelated to the veracity of the message, and those where trust is based on the perceived popular reception of the message. An example of trust based on some perceived reputational aspect of the source is a celebrity source of a message about climate change as opposed to a scientific source. Sterrett et al. (2019) showed that social media news coming from societal elites is more credible than news coming from an established news outlet. An example of trust based on perceived popular reception of the message is the number of “likes” or comments that a message receives. Keselman et al. (2021) found that recommendations by their friends, good reviews and favorable but uncited research increases sharing proclivities. However, Buchanan (2020) found that neither source authority nor evidence of popular engagement with the message influenced sharing proclivities. Harff et al. (2022) found that relationships with online influencers did not make subjects more believing of the influencers’ claims. Avram et al. (2020) found that subjects’ awareness of the quantities of sharing and liking that a message of dubious veracity receives contributes to the risk of believing and spreading it. Ali et al. (2022b) showed that a large amount of “likes” tends to increase the believability of a message. They theorized that a desire to embellish the information explains why people also tend to share such messages. Mena et al. (2020) found that the perceived trustworthiness of a source contributes to the credibility of a false message. Ren et al. (2021) offer a novel perspective. They showed that people can share messages that they are skeptical about because their decision is based on balancing between message veracity and message engagement by others. The prospect of getting lots of likes and comments factors in their decision to share a message of dubious veracity.

### Conspiracy thinking

Anthony and Moulding (2019) found that a conspiratorial worldview conduces to someone’s credence in fake news and that other factors like normlessness relate to credence in fake news through their influence in conspiratorial thinking. On a large-scale study (*N* = 38,113), Kantorowicz-Reznichenko et al. (2022) found that subjects who tend to rely on conspiratorial thinking tend not to change their behavior to align with public health messaging during the Covid-19 pandemic and that people who think deliberately tend not to rely on conspiratorial thinking. This latter finding corroborates Lantian et al. (2021) findings that analytical thinking competency in the argumentative context correlates negatively with a conspiratorial worldview. Calvillo et al. (2021) found that among other factors, subjects who tended to have conspiratorial worldviews tended also to believe false political headlines. How might subjects who rely on conspiratorial thinking tend to be resistant to changing their behavior for public health promotion? Hughes et al. (2022) found a negative correlation between credence in conspiracy ideation and obedience to public health instructions because the subjects perceived low health risk coupled with high risk to livelihood and liberty. Lobato et al. (2020) found that people who scored high on social dominance orientation tend to retransmit disinformation, especially conspiratorial beliefs. Landrum and Olshansky (2019) found that, while conspiratorial and scientific orientations both have significant contributions to credence in scientific disinformation, the contribution of a conspiratorial worldview to resisting scientific knowledge is inconclusive. Miller et al. (2016) found that certain attributes of persons better predict belief in conspiracy theories, namely being well-informed about politics and exhibiting a high level of distrust.

#### Motivated reasoning

“Motivated reasoning” refers to the possibly unconscious exercise of one’s reasoning capacity ostensibly to arrive at a true conclusion, but to achieve some other interest than arriving at the truth. Motivated reasoning has been posited to be an important factor in one’s believing in or endorsing disinformation. A commonly studied form of motivated reasoning is reasoning in order to support one’s political affiliations. Pennycook and Rand (2019) found that subjects’ proclivity to engage in analytical thinking explains their competence at distinguishing true from false headlines even when the false headlines accord with their political affiliations. They conclude that it is inattentive thinking rather than motivated reasoning that contributes to one’s vulnerability to disinformation. Ross et al. (2021) corroborated Pennycook and Rand’s results with a large sample (*N* = 1973) and also found no significant correlation between analytic thinking and a greater inclination to retransmit messages that aligned with one’s political affiliations. Also corroborating Pennycook and Rand, Baptista et al. (2021) found for a sample of Portuguese participants that general political affiliation correlated with susceptibility to disinformation—right-wing participants tended to be more susceptible to left-wing participants, even for disinformation that is discordant with their political affiliation.

However, Calvillo and Smelter (2020) arrived at the opposite result. They found that subjects tended to judge headlines that conflict with their political affiliations as less accurate than headlines that align with their political affiliations, and that subjects that exhibit more analytic thinking were also more biased in their accuracy judgments of the headlines. Others also lend support to the role of motivated reasoning in susceptibility to fake news. Tsang (2021) found that subjects judged identical news information to be false to different levels based on their prior positions on the content, and took this to support the view that motivated reasoning contributes to the retransmission of disinformation. Michael and Sanson (2021) showed that people rely on heuristics that take less effort than analytic thinking in judging the veracity of the news. The bias in their distinguishing true from false headlines is due to the bias in the credence that they place on politically concordant sources rather than the information itself. This finding is corroborated by the findings of Traberg and van der Linden (2022), and goes against earlier findings (Clayton et al., 2019).

Stanley et al. (2022)’s discussion gave some ways in which cognitive capacities involved in assessing truth can also contribute to people’s belief in disinformation, and can shed light on the mechanism linking motivated reasoning to belief in fake news. Vegetti and Mancosu (2020) found that, although people were inclined to perceive politically concordant messages as more believable, people who were well-informed politically can better distinguish true from fake news. With respect to sharing of fake news, Osmundsen et al. (2021) found that political affiliation engenders emotional factors like hatred, which fuels the retransmission of disinformation. Wischnewski et al. (2021) found partial evidence that subjects are more likely to retransmit messages that accord with their own political beliefs and attributed this to motivated reasoning.

### Influential findings

From the 87 curated articles, we looked for those with the most influential findings. We took the number of citations as a proxy for the influence of a paper’s findings. Our inclusion threshold is the threshold number of citations for the 3rd yearly quartile among the articles we curated. Because the number of citations for a paper with influential findings in more recent years can be expected to be fewer than those that have been in the literature for a longer time, we calculated our inclusion threshold on a yearly basis. Based on this method, we included papers with more than 104 citations for 2019, papers with more than 124 citations for 2020, papers with more than 36 citations for 2021, and papers with more than 14 citations for 2022. The increase in the value of the threshold for 2020 over 2019 could be due to interest in pandemic-related disinformation. There was 1 paper each in the years 2016, 2017 and 2018 in our curation, with citation counts of 493, 250 and 1001, respectively. As quartile computation was not possible within these years, we approximated an increase of 1.5 times yearly over the combined 3rd quartile threshold of 2019 and 2020. This yielded the thresholds of 167, 250 and 375 for 2018, 2017 and 2016 respectively. Because they met these thresholds, the 3 papers were included. We had a total of 24 papers. In Appendix 2 consisting of 2 tables, we list the papers and summarize their methodology and findings in the first table. In the second table, we present the papers and the factors that they investigate to display relations between factors.

## Discussion

In this section, we identify five themes from the findings in the previous section and discuss them.

### Anxiety vs. anger

According to Bodenhausen et al. (1994), feeling angry causes subjects to depend more on heuristic cues to arrive at judgment in an argumentative context but sadness causes subjects to depend less on the same cues. Forgas and East (2008) found that being in a bad mood causes one to be more generally disbelieving while being in a good mood causes one to be more vulnerable to being deceived. MacKuen et al. (2010) found that anger encourages heuristic as opposed to systematic reasoning, quickly relying on intuitive cues rather than following a logical order in thinking, while anxious information processing could cause one to entertain contrary perspectives (Martel et al., 2020). In general, the background literature aligns with the studies linking anger with an increased susceptibility to disinformation but appears to oppose the studies linking anxiety with an increased susceptibility to disinformation. This may be due to the possibility that anxiety influences our susceptibility to disinformation jointly with other emotions, beliefs and personality traits, while anger affects our motivation to rely on heuristic cues in judging the truth of information more directly. This possibility is supported by studies of anxiety among a raft of other factors, like risk perception and extraversion.

While the connection between anxiety or anger and cognitive processing like believing has gained wide attention, the connection between anxiety or anger and the other dimension of susceptibility to fake news—retransmitting it—is underexplored. Studies suggest support for the retransmission of fake news via the fact that we are more likely to retransmit what we believe to be true (Buchanan, 2020; Altay et al., 2022), including when we are motivated by altruism (Apuke and Omar, 2021) to help others with what we believe to be true, and believing to be true on the basis of trust (Sterrett et al., 2019; Laato et al., 2020; Melki et al., 2021). However, there appears to be inadequate coverage of mediators of anxiety or anger and retransmission of fake news. Solovev and Pröllochs (2022) found that disinformation spreads more quickly when they contain numerous instances of vocabulary that condemn others. This suggests that people may retransmit what they do not believe to be true in order to express their anger or to punish the perceived perpetrators (see Hartung et al., 2019). Osmundsen et al. (2021) found that hatred may contribute to retransmitting disinformation, which also suggests that punishment is a motivation for retransmission, which may mediate anger and retransmission.

### Identity-protective mechanisms

The anxiety and anger that interfere with judgment may be connected to identity protection. In our literature review of identity, many of the factors studied like political affiliation, bullshitting and overclaiming are indicators of susceptibility to fake news—they correlate with one’s propensity to believe or retransmit disinformation. However, it is unclear what the mechanism linking these factors to identity is. One possibility is that, since these factors relate to one’s promotion and protection of one’s identity, some familiar emotions like anxiety (Wischnewski and Krämer, 2021) or anger mediate between one’s identity and one’s susceptibility to disinformation. Kahan et al. (2017) discuss identity protection. Affinity groups are groups consisting of people who share allegiance to a set of fundamental values. For Kahan, people invest a lot in preserving their positions in affinity groups and the reputation of affinity groups to which they belong. Wischnewski and Krämer (2021) linked Kahan’s thesis to anger and anxiety by postulating that, in encountering information that goes against one’s identity, people will feel angry or anxious, and the emotions will, in turn, cause them to resist the information even if it is true. Van Bavel and Pereira (2018) found that political affiliation can affect recall, tacit assessments and how we perceive. However, identity-protective mechanisms may not exhaust the space of identity as a factor influencing one’s susceptibility to fake news. One might, instead of protecting one’s identity, intend to signal some aspect of one’s identity in conveying information. This hypothesis is compatible with a range of factors influencing one’s susceptibility to spreading disinformation, for example, to signal that one is altruistic. We did not find any research on signaling as a factor in the retransmission of fake news in our curation. The closest was self-promotion (Islam et al., 2020), which can encompass signaling but can also be due to intentions to elicit others’ approval of oneself without going through the recognition that one possesses some valued attribute that is signaled through the retransmission.

### Altruism, communitarianism and emotions

Altruism is one of the factors that receive attention in studies (e.g. Apuke and Omar, 2021). Altruism is a construct that consists of the motivation to benefit others without the expectation of personal recompense. Plume and Slade (2018) document many studies finding altruism as a motivation to disseminate content on social media. It is difficult to tease apart the motivation of pure altruism, communitarian beliefs (Lin, et al., 2022) and associated emotions. For example, a subject may report sharing COVID-19 disinformation on social media out of altruism where the motivator could be a communitarian mindset coupled with anxiety, or a subject could have a desire to share COVID-19 disinformation not from an articulable reason to help others without seeking recompense, but in pursuit of the good feelings that come from perceiving that one has helped others. Studies on communitarian mindsets and susceptibility to the retransmission of disinformation suggest that we should be looking at community-oriented goals and emotions generated in context rather than the general attribute of altruism per se. Given that even momentary priming of subjects to be community-oriented increased their propensity to find meaning in unclear messaging and may increase their propensity to share such messages if they trigger emotions like anxiety (Lin et al., 2022), focusing on altruism as a stable trait may obscure the context-sensitive nature of the attribute.

### Bias triggers vs. emotions

The studies linking greater susceptibility to disinformation with repeated exposure to the disinformation suggest that there can be non-emotional distractors from thinking analytically. The link between repeated exposure and belief is well-documented in studies about the illusory truth effect. This is the phenomenon of finding information truer with repeated exposure to it. The phenomenon was first identified in Hasher et al.’s (1977) study where students were more likely to rate sentences as “true” after repeated exposures to them. Hassan and Barber (2021) survey the research in the context of disinformation. Studies on the continued influence effect show that the effect persists even in the face of correction of false information. A recent overview of research on the effect can be found in Kan et al. (2021). The illusory truth effect and the continued influence effect could account for “falling for fake news” in the sense of explaining why subjects believe fake news. However, Swire et al. (2017) showed that repeated exposure to a myth in corrections only served to slow down believing the correct information. They did not find evidence of the myth-repeating correction backfiring to promote belief in the myth. While the bulk of the literature pertains to believing disinformation, Effron and Raj (2020) study suggests that repeated exposure can also affect one’s motivation to retransmit disinformation, in their case, by decreasing the perceived ethicality of retransmitting disinformation headlines. Stepping back, we observe that repeated exposures differ from emotions in that they may render us susceptible to disinformation without involving emotions, yet also differ from those factors that operate within reasoning in that they do not appear to feature in reasoning about the truth. They may be simply tendencies we have that are triggered by our exposure to the world.

### The “saving” function of analytical thinking

The literature about the significance of analytical thinking to susceptibility to fake news overwhelmingly relates analytical thinking to truth discernment (e.g. Martel et al., 2020; Pennycook and Rand, 2019, 2020; Lantian et al., 2021; Ross et al., 2021). From here, if there is a link to the spreading of fake news, it is conceivably by way of the fact that we find the information true and want to inform others of the truth, or that we are not stopped from spreading the information by finding it false. This suggests the assumption that analytical thinking has a “saving” function—the exercise of analytical thinking facilitates one’s distinguishing truth from falsity and “saves” us from believing and/or spreading false information. Associated with the assumption is the idea that the better one is at analytical thinking or the more one is disposed to employ analytical thinking, the better one is at using the right heuristics to defend against the threat of fake news, for example, to correctly rely on scientific sources of information, to appropriately assess that social endorsement is relevant, and to accurately fact-check.

Pit against these ideas is the view that analytical thinking is a double-edged sword. It may facilitate credence in and spread of disinformation as much as it may prevent them. In this section, we consider three points that mitigate the significance of analytical thinking as a contributing factor in truth discernment and the retransmission of fake news. The first is that biases could operate in the analytical thinking of someone who does well in analytical thinking measures. The second is that someone who is good at analytical thinking or disposed to think analytically may also be good at or disposed to exercise motivated reasoning. The third is that good reasoning need not be directed at seeking the truth. Before that, let us take a closer look at how factors serve as distractions in analytical thinking and the implications of such roles.

#### Biases that operate in analytical thinking

Confirmation bias has been proposed to make us more effective persuaders by helping us recruit information relevant to supporting our standpoints (Mercier and Sperber, 2011), contribute to the rigor and comprehensiveness of a group’s deliberation when its members exert themselves in defending their standpoints (Smith and Wald, 2019), facilitate group coordination by reducing intentions of group members that are incompatible with the rest of the group (Norman, 2016), and to effect social change in order to match our beliefs (Peters, 2022). Having a bias towards treating as true information that derives from the right types of sources (peer-reviewed scientific journals, mainstream news platforms, domain-relevant experts) can save us time and effort in fact-checking every piece of information that comes our way. Similarly, ideally, user ratings or opinions that are based on a large and varied enough sample and not affected by other biases of the raters (see de Langhe et al., 2016; Godden, 2008) should enable consumers to judge the goodness of a product or the truth of information without having to do further research. Conspiracist thinking allows us to detect true conspiracies and defend ourselves against them (van Prooijen and van Vugt, 2018).

Ideal analytical thinking proceeds by way of careful reasoning—whether conducted between interlocutors or with oneself in analytical reflection—and is aimed at uncovering the truth. Reasoning consists of giving reasons for claims, which summarize premises for conclusions. However, making and evaluating arguments is cognitively demanding and time-consuming. To make thinking more efficient, we rely on mental heuristics. While they may usually serve us well, mental heuristics can also cause us to believe in disinformation (see Ali and Zain-ul-abdin, 2021; Brashier and Marsh, 2020). Confirmation bias, source credibility, social endorsement and conspiracist thinking can interfere with the course of ideal analytical thinking. They can cause us to accept information as true or they can cause us to judge information as true on their bases, and to use the information as reasons for our claims. They can cause us to skip steps in evaluating the reasons for a claim or to uncritically accept claims. Because these biases operate within the exercise of analytical thinking, tests for a general propensity or competence to think analytically may not detect their operation. One may be predisposed to engage in analytical thinking or be good at the tested type of analytical thinking (for example, traditional CRT’s numerical analytical thinking) but have their reasons and claims subject to interference by biases. Biases that operate within analytical thinking, then, can undermine hypotheses that analytical thinking guards us against falling for fake news. Due to the operation of biases in analytical thinking—in the selection of reasons for claims, the weight accorded to reasons and the requirement for rigor in reasoning—one may fall for fake news even when one is predisposed to thinking analytically.

#### Inconclusiveness about motivated reasoning’s role

Pennycook and Rand (2019) argued that it is being distracted from thinking analytically, rather than motivated reasoning, that causes us to fall for fake news. Their claim is based on their findings that, regardless of content alignment with subjects’ beliefs, subjects’ competence in analytical thinking as measured by the CRT correlated positively with truth discernment. The motivated reasoning thesis would predict positive correlations between high analytical thinking competency scores and truth discernment accuracy when discerning the truth of content that is aligned with subjects’ beliefs, and a negative correlation between high competency scores and truth discernment accuracy when discerning the truth of content that are not thus aligned.

There is substantial work on analytic thinking and its effect on believing fake news that is aligned with Pennycook and Rand’s (2019) conclusions in undermining the hypothesis that our believing fake news is due to motivated reasoning (e.g. Pennycook and Rand, 2020; Clayton et al., 2019; Martel et al., 2020; Wischnewski and Krämer, 2021). Nevertheless, earlier work on motivated reasoning for topics like public discourse (Kahan et al., 2017) and misinformation (Kahan, 2017), together with suggestive work on confirmation bias (Zhou and Shen, 2022) and possibly opposing conclusions from recent work (Calvillo and Smelter, 2020; Tsang, 2021; Michael and Sanson, 2021; Traberg and van der Linden, 2022) urges caution in jettisoning the motivated reasoning research on fake news believability.

There are two points standing in the way of downplaying the causal relevance of motivated reasoning to believing disinformation. First, subjects who are good at analytical thinking may engage in motivated reasoning only when identity or reputational stakes are high. This would occur in a setting where one has to defend one’s position to others, rather than answer analytical questions in private and anonymously. Pennycook and Rand admit that their study could have different results if the questions were less factual and more personal (Pennycook and Rand, 2019). Second, the CRT items—analytical questions of “brain teaser” variety— may prime subjects to focus on exercising their analytical thinking abilities to the exclusion of the factors that typically motivate motivated reasoning.

### Non-truth-directed reasoning to spread fake news

Non-truth-directed reasoning may cause individuals to retransmit fake news despite scoring well on analytical thinking measures. Reasoning need not aim at evaluating truth. Some researchers distinguish between “accuracy-oriented” and “goal-oriented” reasoning (for example, Osmundsen et al., 2021), where the former aims at the truth while the latter does not. Goal-oriented or means-ends reasoning can lead one to accept the truth of content that one is unsure of. This may occur in the minds of anxious individuals debating whether to believe in some potentially life-saving remedy in the panic and confusion at the beginning of the Covid-19 pandemic. Or, they may also reason to the conclusion that they should retransmit the information despite being uncertain about its truth. Similar means-ends reasoning can be conducted rigorously for deciding to retransmit unverified information for the sake of eliciting social engagement (likes, comments, ratings), to signal political affiliation, convey moral outrage, inflict social punishment, and/or to protect one’s identity or enhance one’s reputation in some other way. More perniciously, quasi-“truth directed” reasoning can cause people to classify information that they are unsure about, or that they believe is false, as “interesting if true (Altay et al., 2022)” or “likely to become true” (Helgason and Effron, 2022). As research has shown, such classifications suffice to motivate the retransmission of the relevant information.

## Conclusions

To our research question “What psychological distractions to analytical thinking mediate the belief in and/or retransmission of fake news?”, we have identified the following distractions by reviewing the research literature from 2016 to 2022: Emotions (especially anxiety and anger), repeated exposure, altruism, identity protection or enhancement, confirmation bias, source credibility, social endorsement, conspiracy thinking and possibly motivated reasoning. We think that Pennycook and Rand (2019) are generally correct when they say that people fall for fake news because they are distracted from thinking. However, their view can be qualified by these five conclusions from our discussion.

First, it is not analytical thinking per se, but analytical thinking directed to evaluating the truth that safeguards us from believing or spreading fake news. Someone may be good at analytical thinking and employ it in means-ends reasoning to decide to accept information that one is uncertain about as true, or to spread information that one knows is false in order to achieve their purposes. Some means-ends reasoning masquerade as truth evaluations—quasi-“truth directed” reasoning like “interesting if true” or “likely to become true” may inform one’s decision to believe the information.

Second, psychological factors can distract us from exercising analytical thinking and they can also distract us in exercising analytical thinking. They distract us from analytical thinking when, for example, we find a statement true because we were exposed many times to it. They distract us in analytical thinking when we favor certain premises in our reasoning because they support what we already believe. When we believe fake news that aligns with our political orientation, do we believe as a result of reasoning to confirm what we already believe or do we believe as a result of being triggered by prior exposure? The former is a bias that distracts within analytical thinking while the latter is a bias that distracts from analytical thinking.

Third, whether a psychological factor functions as a distractor within analytical thinking or from analytical thinking may depend on contextual factors. For example, under what conditions do we deliberate about source credibility in order to make an accuracy judgment and under what conditions do we make a snap accuracy judgment based on source credibility? The same heuristics that facilitate reasoning or are adaptive in some way can cause us to believe or retransmit disinformation. This suggests that investigating contextual factors influencing the choice of heuristics or how a heuristic is deployed is a potentially fruitful line of research.

Fourth, due to a possible mismatch between the type of analytical thinking that tests of analytical thinking employed by many studies on analytical thinking and disinformation employ and the type of analytical thinking that informs one’s decision to believe or spread information, measurements of analytical thinking may not indicate vulnerability to believing or spreading fake news. Many disinformation studies that seek to measure analytical thinking ability rely on CRT. Might relevant dimensions of analytical thinking ability be missed out by the CRT? Dimensions pertaining to argumentation—assessing reasons for claims, evidence for reasons, and effectiveness of objections and counterexamples—seem relevant to the persuasiveness of fake news. These dimensions are not well served by the CRT and only one study (Lantian et al., 2021) in our curation investigated them.

Fifth, despite the influential Pennycook and Rand (2019) and substantial other research aligned with its skepticism about the role of motivated reasoning to fake news susceptibility, the relevance of motivated reasoning to our tendency to believe fake news should not yet be dismissed. The conditions under which people undertake motivated reasoning (for example, only when faced with an imminent threat to one’s identity or reputation) and the priming effects of analytical tests on accuracy assessments of true and false information have not been adequately investigated. For the latter, it is not only the CRT but any analytical test could prime subjects to treat tasks in the immediate future as analytic ones, requiring objective and careful reasoning. This could preclude the operation of possible emotional triggers of motivated reasoning like anger or anxiety.

What is the outlook for research on psychological factors affecting reasoning and disinformation? With the advent of disinformation fabricated by artificial intelligence right down to proper citations of fabricated sources, the arms race against disinformation is intensifying. The identification of psychological factors that can distract us away from reasoning or distract us within reasoning is not an end in itself. It is for the purpose of education and public messaging that can deflect at least the most pernicious of fake news disinformation influences. The five conclusions above correspondingly suggest the following topics of research that can contribute to the design of education and public messaging initiatives:

1. Truth-oriented values (e.g. intellectual integrity, open-mindedness, intellectual humility) and how they might serve as constraints to goal-oriented reasoning, as opposed to accuracy-oriented reasoning
2. Distractors away from reasoning and distractors within reasoning from among the extant psychological factors that influence our vulnerability to disinformation (especially in untangling the potentially conflicting roles of anxiety and worry)
3. Conditions under which a psychological factor distracts us away from reasoning and conditions under which it distracts us from reasoning
4. Measures of other dimensions of analytical thinking (especially argumentative reasoning) to gauge the analytical thinking factor influencing our vulnerability to disinformation
5. Topics concerning motivated reasoning (especially high-stakes motivated reasoning as a factor influencing our vulnerability to disinformation, the role of emotion in motivated reasoning causing us to believe/spread fake news, and conceptual work distinguishing confirmation bias from motivated reasoning)

Finally, while it is not within the ambit of our research question, we noticed that there is little work done on the relation between believing and spreading fake news. The studies that we curated understand “falling for fake news” or “vulnerability to fake news” in terms of believing it, spreading it, or both. The propensity to believe and the propensity to spread fake news are usually taken as dependent variables on other factors but not on one another. Yet, these two propensities practically exhaust our understanding of what it is to be vulnerable to fake news. It is therefore important for our taming of fake news to learn how belief in it interacts with its retransmission.

## Supplementary information

Distractions, Analytical Thinking and Falling for Fake News: A Survey of Psychological Factors
