# Supplementary material for: Distractions, analytical thinking and falling for fake news: A survey of psychological factors
Source: Humanit Soc Sci Commun. 2023 Jun 12;10(1):319. doi: 10.1057/s41599-023-01813-9 (PMC10259813; doi:10.1057/s41599-023-01813-9)
Supplement: Supplementary file 1 — Distractions, Analytical Thinking and Falling for Fake News: A Survey of Psychological Factors [file 41599_2023_1813_MOESM1_ESM.docx]

Article title:

Distractions, Analytical Thinking and Falling for Fake News: A Survey of Psychological Factors

Authors:

Adrian Kwek

Luke Peh

Josef Tan

Lee Jin Xing

Appendix 1

Notes

- “Falling for fake news” consists in either believing fake news or spreading fake news.
- The direction of arrows indicates positive or negative causal relevance suggested by the literature review.

Of celebrities

Of quantity or quality of reviews

Of message engagement

Of personal contacts

Of scientific authority

Of traditional media credibility

Of social media

Believing fake news

Source credibility

Spreading fake news

Repeated exposure

Motivated reasoning

Identity

Altruism

Conspiracy thinking

Confirmation bias

Social endorsement

Authoritativeness

Self-promotion

Religiosity

Collectivism

Sadness

Emotions

Anger

Worry

Anxiety

Appendix 2

Appendix 2 consists of Table 1 and Table 2. Table 1 lists the influential articles obtained by the method described in Summary of Influential Findings, with respect to article author(s) and year, methodology, findings, factors investigated and their relevance to the paper. Table 2 presents a visual comparison of the topics of relevance covered by each paper.

Table 1

| Article | Methodology | Findings | Factors | Relevance |
| --- | --- | --- | --- | --- |
| Altay et al. (2022) | Participants rated news accuracy, interestingness-if-true and willingness to share. (N = 904) | 1. Willingness to share had positive association with interestingness-if-true ratings.  2. Willingness to share had positive association with accuracy perceptions.  3. Fake news had lower accuracy ratings than true news, and had higher ratings for willingness to share.  4. Trust in mass media was positively associated with correct accuracy ratings.  5. Particularly for true news, prior exposure was associated positively with higher accuracy ratings. | Dependent variables (DV)  Sharing, accuracy  Independent variables (IV)  Interestingness-if-true, trust in mass media, prior exposure | Source credibility, repeated exposure, sharing, accuracy |
| Apuke et al. (2021) | Participants rated information sharing, social interaction gratification, entertainment, altruism, information seeking, pass time and fake news sharing (N = 385) | 1. Altruism strongly predicted fake news sharing.  2. Information sharing, social interaction gratification, information seeking and pass time predicted fake news sharing.  3. There was no significant association between entertainment and fake news sharing. | DV  Sharing  IV  Altruism, information sharing, social interaction gratification, information seeking, pass time | Altruism, sharing |
| Buchanan (2020) | Participants were evaluated on their performance on digital literacy, personality and political orientation by completion of relevant scales. Simulated ‘news’ items were given contrasting authoritativeness by source attributions. Participants rated their likelihood to propagate the ‘news’ items. (N = 2634) | 1. People who believe disinformation to be true, have beliefs that are aligned with the disinformation or are familiar with the disinformation are more likely to spread it.  2. People who are younger, male, less educated are more likely to spread disinformation.  3. People who score low in Agreeableness and Conscientiousness, and high in Extraversion and Neuroticism, are more likely to spread disinformation.  4. Alignment and familiarity are strongly correlated with likelihood of spreading disinformation; the other factors are weakly correlated.  5. Perception of consensus, level of digital literacy and source authoritativeness were not shown to be correlated with likelihood of spreading disinformation. | DV  Sharing  IV  Alignment, age, gender, education level, agreeableness, conscientiousness, extraversion, neuroticism, familiarity, perception of consensus, digital literacy, source authoritativeness | Repeated exposure, confirmation bias, source credibility, social endorsement, sharing |
| De Coninck et al. (2021) | Participants from 8 countries were surveyed about their belief in conspiracy theories, misinformation, information sources, trust in the sources, level of anxiety, level of depression, and demographic attributes. (N = 8806) | 1. Exposure to healthcare professionals was largely negatively associated with conspiracy and misinformation beliefs, while exposure to political personalities was positively associated with conspiracy beliefs in some countries and with misinformation beliefs all.  2. Exposure to traditional media tended to be negatively associated with conspiracy and misinformation beliefs, but the results were not consistent between countries.  3. Trust in healthcare professionals tended to be negatively associated with conspiracy and misinformation beliefs.  4. Obtaining information from healthcare professionals tended to be negatively associated with conspiracy and misinformation beliefs, while obtaining information from digital media together with high trust levels in them tended to be positively associated with conspiracy and misinformation beliefs.  5. Anxiety did not tend to be associated with conspiracy and misinformation beliefs except for one country, wherein it was negatively associated with conspiracy and misinformation beliefs.  6. Depression was strongly associated with conspiracy and misinformation beliefs. | DV  Belief in conspiracy theories, belief in misinformation  IV  Information sources, trust in information sources, anxiety, depression | Emotions, source credibility, conspiracy thinking |
| Hughes et al. (2022) | Participants completed scales on compliant behaviors, preventive behaviors, risk perceptions, belief in conspiracy theories, together with demographic questions. (N = 368) | 1. Having conspiracy beliefs was negatively associated perceptions of risk to own and others’ health.  2. Having conspiracy beliefs was positively associated with perceptions of economic, liberty and informational risks.  3. Having conspiracy beliefs and complying with health regulations was mediated by perception of health, economic and liberty risks. | Belief in conspiracy theories,  perception of risk to own health, others’ health, economy, liberty, information; complying with health regulations | Emotions, conspiracy thinking |
| Islam et al. (2020) | Participants were surveyed on their use of social media with respect to self-promotion, entertainment, religiosity, deficient self-regulation, exploration, social media fatigue and unverified information sharing, together with demographic questions. (N = 433) | 1. Deficient self-regulation and self-promotion were significantly positively predictive of unverified information sharing.  2. Exploration was significantly negatively predictive of unverified information sharing.  3. Entertainment and social media fatigue were slightly positively predictive of unverified information sharing.  4. Deficient self-regulation and exploration were significantly positively predictive of social media fatigue.  5. Entertainment was slightly negatively predictive of social media fatigue. | Self-promotion, entertainment, religiosity, self-regulation, exploration, social media fatigue, unverified information sharing | Identity, sharing |
| Laato et al. (2020) | Participants were surveyed on trust in online information, information overload, perceived severity of Covid-19, perceived susceptibility to Covid-19, anxiety from online health searches, and unverified information sharing, together with demographic questions. (N = 294) | 1. Trust in online information and information overload were significantly positively predictive of unverified information sharing.  2. Perceived susceptibility to Covid-19 and perceived severity of Covid-19 were not significantly predictive of unverified information sharing.  3. Trust in online information and information overload were significantly positively predictive of anxiety from online health searches, information overload more so than trust in online information.  4. Females had a greater tendency to experience anxiety from online health searches, but a smaller tendency to share unverified information than males. | Trust in online information, information overload, perceived severity of Covid-19, perceived susceptibility to Covid-19, anxiety from online health searches, and unverified information sharing | Emotion, source credibility, sharing |
| Lantian et al. (2021) | Participants were assessed on their critical thinking ability using an open-ended test, and their belief in conspiracy theories measured on a Conspiracy Belief Scale. Study 1 (N = 86) served to establish initial association. Study 2 (N = 252) served to assess the contribution of subjective self-perceptions of critical thinking ability to belief in conspiracy theories on a wider sample. | Study 1  1. Critical thinking ability was slightly negatively associated with belief in conspiracy theories.  Study 2  1. Critical thinking ability was negatively associated with belief in conspiracy theories.  2. There was no evidence of association between subjective self-perceptions of critical thinking ability and belief in conspiracy theories. | Critical thinking ability, belief in conspiracy theories, self-perception of critical thinking ability | Conspiracy thinking |
| Lin, et al. (2022) | The paper presents 7 studies on collectivism and finding empty claims meaningful.  Study 1  Data from the General Social Survey (USA) was analyzed with respect to responses concerning belief in astrology and collectivism. (N = 5114)  Study 2  Data from the Chinese Online Social Survey (China) was analyzed with respect to responses concerning belief in fortune-telling, palm-reading, Feng Shui, a pseudoscientific report and collectivism. (N=9638)  Study 3  Participants were surveyed with respect to collectivism and a) false memories of fabricated fake news and belief in existing fake news (N = 193), b) belief in Covid-19 fake news, sharing propensity, and measures of positive and negative emotions (N = 200), and c) belief in non-Covid-19 fake news (N = 200).  Study 4  Participants from USA (N = 122) and China (N = 318) were surveyed about collectivism and their propensity to find vague claims meaningful, agreement with verifiable statements on daily life, belief in astrology, agreeableness and thinking style.  Study 5  Participants rated empty claim items and recorded their thoughts upon exposure to the items. They were also assessed for collectivism levels. (N = 250)  Study 6  Participants were induced using a force-agreement paradigm to self-perceive as collectivistic or not collectivistic momentarily. They then rated the profundity of vague sentences in one study and the informativeness, meaningfulness and persuasiveness of a piece of fabricated fake news in another study. (N = 300)  Study 7  Participants were asked to rate the meaningfulness of metaphorical-sounding sentences after being led to infer that the sentence comes from a human or informed that the sentence does not. (N = 119) | Study 1  Collectivism was positively associated with finding astrology scientific and believing a pseudoscientific statement.  Study 2  Collectivism was positively associated with belief in fortune-telling, palm-reading, Feng Shui and the pseudoscientific report.  Study 3  1. Collectivism was positively associated with believing fabricated Covid-19 fake news, but not with believing existing Covid-19 fake news.  2. Collectivism was positively associated with believing Covid-19 fake news and with sharing the fake news.  3. Collectivism was positively associated with believing non-Covid-19 fabricated fake news.  Study 4  1. Chinese participants perceived vague claims as more meaningful than American participants.  2. Participants who endorsed collectivistic values were more likely to find vague claims meaningful.  3. Collectivism effects were not due to agreeing with verifiable statements on daily life, agreeableness or holistic thinking.  Study 5  Higher collectivism was associated with finding the empty claim items more meaningful.  Study 6  Participants who were induced to momentarily self-perceive as collectivistic tended to rate vague sentences as more profound and find the fabricated fake news more informative, meaningful and persuasive than participants who were induced to momentarily self-perceive as not collectivistic.  Study 7  There was a positive association between collectivism and experience of meaningfulness when participants were under the impression that the sentence came from a human than when they were not. | DV  Finding empty claims meaningful  IV  Collectivism, endorsement of collectivistic values, momentary self-perception as collectivistic, perception of human source | Altruism, identity |
| Martel et al. (2020) | The article presents 2 studies about the contribution of emotion to belief in fake news.  Study 1  Participants were surveyed on their current emotions using the Positive and Negative Affect Schedule scale and then rated the accuracy of news or fake news headlines categorized as republican or democratic. (N = 409)  Study 2  Participants were assigned to 1 of 3 groups: induced to use reason, induced to use emotion, and control group. They were then required to judge the accuracy of news or fake news headlines categorized as republican or democratic. (N = 3884) | Study 1  1. The level of current positive or negative emotions, except for those emotions associated with analytical thinking, was positively associated with belief in fake news and negatively associated with truth discernment.  2. Alignment of information with political views was not a significant mediator between emotion and accuracy judgments.  Study 2  1. Reliance on reason was positively associated with rating fake news as less accurate, and generally with truth discernment.  2. Reliance on reason was positively associated with belief in real news, but not associated with belief in fake news.  3. Reliance on emotion was positively associated with belief in fake news but not real news.  4. There was no significant association between reliance on reason and alignment of information with political views. | DV  Truth discernment, belief in fake news  IV  Positive emotions, negative emotions, alignment with political views, reliance on reason, reliance on emotion | Emotions, confirmation bias, motivated reasoning, accuracy |
| Melki et al. (2021) | Participants were surveyed by telephone on media trust, media exposure, belief in myths, conspiracies and false information, social media posting practices, media literacy and demographics. (N = 792) | 1. Trust in social media, interpersonal communication and clerics as news sources was positively associated with belief in fake news.  2. Trust in government information sources and education level were negatively associated with belief in fake news.  3. Belief in fake news is negatively associated with verifying information before sharing on social media.  4. Only media literacy is positively associated with verifying information before sharing on social media. | DV  Belief in fake news, sharing on social media  IV  Trust in social media, interpersonal communication, clerics; trust in government information sources, education level | Source credibility |
| Miller et al. (2016) | Two datasets were analyzed. The first is from an original survey of participants (N = 2203) and the second is from the 2012 American National Election Study (N = 2485).  Participants were analyzed with respect to the following factors: conspiracy endorsement (of beliefs categorized as favorable to conservatives or liberals), ideology (from extremely conservative to extremely liberal), knowledge about politics, and trust. | 1. Conservatives are more likely to have ideologically-motivated conspiracy endorsement than liberals.  2. Having knowledge mediates ideologically-motivated conspiracy endorsement, but only for conservatives.  3. Having knowledge and trust is positively associated with ideologically-motivated conspiracy endorsement, but only for conservatives. | DV  Ideologically-motivated conspiracy endorsement  IV  Political ideology, knowledge about politics, trust | Confirmation bias, source credibility, conspiracy thinking |
| Osmundsen et al. (2021) | Twitter participants were surveyed on hypothesized accuracy-oriented and goal-oriented predictors of fake news sharing: ignorance (tested by the Cognitive Reflection Test, factual political questions and a digital media literacy test), disruptive motivations (trolling and political cynicism), and political polarization. With their permission, the participants’ Twitter accounts were scraped for actual sharing of real and fake politically slanted news. (N = 2337) | 1. Fake news sharing is confined to a small part of the Twitter user population.  2. Republicans have a greater tendency than Democrats to propagate fake news and fake news sources.  3. Participants who politically identified with a news source had a greater tendency to propagate news from that source. | Ignorance (CRT scores, factual political knowledge, digital media literacy), disruptive motivations (trolling, political cynicism), political polarization, actual sharing of real and fake politically slanted news. | Sharing |
| Pennycook & Rand (2019) | The paper presents 3 studies on analytical thinking vs. motivated reasoning on fake news accuracy judgments. (N = 3446)  Study 1  Participants were presented with headlines together with byline, picture and source. These were categorized as Republican-consistent, Democrat-consistent or politically neutral. Participants rated these items according to how accurate they thought the items were, whether they would share it online, and whether they had seen it before. They also had to complete 2 versions of the Cognitive Reflection Test (CRT), a measure for pseudo-profound bullshit receptivity, and demographic questions.  Study 2  This study attempted to replicate the findings of Study 1 with more news items and participants.  Study 3  This study attempted to gauge the effect of implausibility on accuracy judgements by comparing with pretest-derived item plausibility and partisanship scores. | 1. There was no evidence that CRT scores had positive association with more politically-aligned news items being judged accurate.  2. There was no evidence that CRT scores had positive association with propagation of fake news on social media.  3. Higher CRT scores were associated with rating fake news as less accurate independently of whether the news is politically aligned with the rater.  4. Higher CRT scores were mostly associated with rating real news as more accurate.  5. Participants who tended to rely on analytic thinking were better able to distinguish fake and real news items.  6. Participants tended to be better able at distinguish fake and real news items that were aligned with their political ideologies than not.  7. Participants with higher CRT scores tended to judge as inaccurate items with lower plausibility scores, and tended to judge as accurate items with higher plausibility scores, irrespective of partisanship. | DV  Accuracy, truth discernment, sharing  IV  Reliance on analytic thinking (CRT scores), partisanship, alignment with political ideology, plausibility | Motivated reasoning, accuracy, sharing |
| Pennycook et al. (2018) | The paper presents 3 studies on the effects of repeated exposure, implausibility, warnings and on the perceived accuracy of fake news.  Study 1  Participants rated the interestingness and accuracy of extremely implausible false statements, known true statements, statements that are obscure but true, and statements that are obscure but false, together with demographic questions. (N = 409)  Study 2  Participants were divided into a group that were given the news items together with a warning accompanying the fake news items, and a control group without the warnings. They rated whether they would share the news items. Then they completed demographic and political partisanship items. Finally, they rated the familiarity and accuracy of a larger set of news items including the first items. (N = 949)  Study 3  Participants underwent a similar study design as Study 2, with an increased filler stage and a follow-up session after a week. At the familiarization stage, participants were asked to rate the items on familiarity rather than propensity to share on social media. (N = 940) | Study 1  Repeated exposure was positively associated with perceptions of accuracy, except for extremely implausible statements.  Study 2  A one-time exposure to the same news item is positively associated with perceived accuracy, irrespective of real or fake news, when the fake news carries a warning at the first exposure, irrespective of whether the news content is aligned with one’s political ideology, and irrespective of whether the participants are conscious of the first exposure.  Study 3  Prior exposure to a fake news item is positively associated with its perceived accuracy, the effect lasts even after a week, and has lasting increases with more exposures. | DV  Perceived accuracy of fake news  IV  Plausibility, repeated exposure, political alignment | Repeated exposure, motivated reasoning, accuracy |
| Pennycook et al. (2020) | The paper presents 3 studies on the psychological profiles of people who are susceptible to believing fake news.  Study 1  Participants rated fake news headlines for accuracy, and completed measures for analytical thinking, pseudo-profound bullshit receptivity (propensity to consider randomly produced sentences profound) and overclaiming (claimed familiarity with a fabricated item). They also answered demographic questions. (N = 401)  Study 2  In addition to the items in Study 1, participants rated real news, prototypically profound statements, and half of the participants were not exposed to the sources of information. (N = 402)  Study 3  The study is an analysis of responses to of bullshit receptivity and familiarity measures of a dataset from a completed study. (N = 802) | 1. Pseudo-profound bullshit receptivity has a positive association with judging fake news to be accurate.  2. Pseudo-profound bullshit receptivity has a negative association with distinguishing real from fake news.  3. Overclaiming has a positive association with judging fake news to be accurate.  4. Analytic thinking has a negative association with judging fake news to be accurate, and is not moderated by news source awareness or headlines familiarity. | DV  Accuracy, truth discernment  IV  Pseudo-profound bullshit receptivity, overclaiming, analytic thinking, source awareness, familiarity | Repeated exposure, source credibility, accuracy |
| Ross et al. (2021) | Participants were presented with a set consisting of real news, fake news and true (“hyperpartisan”) news that is reported in a politically biased way. They either rated the news items for accuracy or their willingness to share. They also completed measures of analytical thinking, political orientation and demographics. (N = 1973) | 1. There was no evidence for a positive association between analytic thinking scores and accuracy judgments of politically aligned hyperpartisan or fake news.  2. There was a negative association between analytic thinking scores and accuracy judgments of politically aligned hyperpartisan or fake news.  3. There was largely evidence for a positive association between analytic thinking scores and ability to distinguish true from false news, irrespective of political alignment. | DV  Accuracy, truth discernment, sharing  IV  Analytic thinking, political alignment | Confirmation bias, motivated reasoning, accuracy, sharing |
| Sterrett et al. (2019) | Participants were presented with the same fabricated news item, but accompanied with different named sharers and information sources. Some are trustworthy public figures and news platforms. Participants answered questions on trust in the article, engagement (including propensity to share), trust in the sharer, trust in the information source, and demographic questions. (N = 1489) | 1. Trust in a news item is positively associated with trust in its sharer.  2. Engagement with a news item, e.g. sharing it or its source, is positively associated with trust in its sharer.  3. Engagement with a news item is positively associated with trust in its source, but less than with trust in its sharer.  4. There is no significant association between trust in a news item and whether its source is fabricated or trusted.  5. Interest in the subject matter and obtaining the news on social media are positively associated with trust and engagement. | DV  Trust in article, propensity to share article or source  IV  Trust in source, trust in sharer, interest in subject matter, news source | Source credibility |
| Su (2021) | Participants were surveyed on the following factors: social media use, discussion heterogeneity preference (openness to political differences in others), worry about being infected by Covid-19, faith in scientists, belief in lab misinformation, belief in vaccine misinformation, demographic information and political orientation. (N = 3080) | 1. Social media usage was positively associated with belief in misinformation.  2. Discussion heterogeneity preference was negatively associated with belief in misinformation.  3. Worry increased the positive association between social media use and belief in misinformation.  4. Worry increased the negative association between discussion heterogeneity preference and belief in misinformation.  5. Faith in scientists increased the effect of worry on social media use and belief in misinformation; and on discussion heterogeneity preference and belief in misinformation. | Social media use, discussion heterogeneity preference (openness to political differences in others), worry about being infected by Covid-19, faith in scientists, belief in lab misinformation, belief in vaccine misinformation, demographic information, political orientation | Emotions, source credibility |
| Swire et al. (2017) | The paper presents 2 experiments about the role of familiarity in correcting inaccurate information.  Experiment 1  Participants read myths and facts, which were labelled as such. The myths were then debunked, with the explanations repeating the myth. They also had to answer inference questions. (N = 93)  Experiment 2  This experiment was similar to the first, except for having older adult participants where the first had undergraduates, and having a 3-week retention period. (N = 109) | 1. Revision of belief was preserved for a longer duration after fact affirmation (familiarity aligns with recollection) than after myth retraction (familiarity conflicts with recollection).  2. Familiarity can affect inferences.  3. Elderly participants (65 and older) are worse in retaining their beliefs that the myths are inaccurate after receiving the corrections. | DV  Corrected beliefs about misinformation  IV  Fact affirmation, myth retraction, familiarity, age | Repeated exposure |
| Traberg & van der Linden (2022) | The paper presents a pilot and a main study.  Pilot study  Participants was surveyed on their perception of political bias of news sources, their own political affiliations, and their judgments about the reliability of misinformation. (N = 656)  Main study  Participants rated the accuracy of headlines, their likelihood of sharing, and credibility and political bias of news sources. In addition, they answered demographic questions. (N = 150) | 1. The political alignment of a news source with one’s own political affiliation negatively correlated with one’s likelihood of getting accuracy judgements about the factual information or misinformation wrong, irrespective of political affiliation.  2. Being liberal was positively correlated with one’s likelihood of getting accuracy judgments about factual information from news sources that were not politically aligned with one’s affiliation wrong.  3. The effects of political alignment of news sources on accuracy judgments are mediated by how credible the sources were perceived to be. | DV  Accuracy, sharing  IV  Message alignment, political affiliation, source credibility | Confirmation bias, source credibility, motivated reasoning, accuracy, sharing |
| Xiao et al. (2021) | Participants were surveyed on social media news use, social media news trust, misinformation identification (self-reporting of how frequently one detects misinformation online), Covid-19-related conspiracy beliefs (agreement with statements), general conspiracy (agreement with statements) beliefs, gender, age and party affiliation. (N = 760) | 1. Social media news use was positively associated with belief in general and Covid-19 conspiracy theories.  2. Social media news trust increased the positive association between social media news use and belief in conspiracy theories.  3. Misinformation identification was decreased the positive association between social media news use and belief in conspiracy theories when social media news trust was low. | Social media news use, social media news trust, misinformation identification, Covid-19-related conspiracy beliefs, general conspiracy beliefs, gender, age, party affiliation | Source credibility, conspiracy thinking |
| Zhou & Shen (2022) | Participants were screened into a group believing that climate change caused by humans is real and another that believes it is a hoax. They are then surveyed for their demographic information, rated the certainty of their climate change beliefs at screening stage, and watched 3 videos with a climate change message in random order. After each video, they rated the factuality, trustworthiness of source, empathy and effectiveness of the message. After all the videos, they rated their climate change attitude and preference of climate change public policies. (N = 408) | 1. The alignment of prior belief with message content was more positively associated with credibility (expertise and trustworthiness) than non-alignment.  2. The alignment of prior belief with message content was more positively associated with the perception that the message content was factual than non-alignment.  3. Empathy level was more positively associated with messages whose content was aligned with prior belief than those that were not aligned.  4. Message effectiveness was more positively associated with messages whose content was aligned with prior belief than those that were not aligned. | DV  Credibility (expertise and trustworthiness), perception of factuality, empathy, perception of message effectiveness  IV  Alignment of prior belief with message content | Source credibility, confirmation bias |
| Zimmerman et al. (2020) | In 3 waves across 2 months, participants rated the familiarity and truthfulness of headlines that were a mix of real news, actual fake news and fabricated fake news. They also completed measures on trust in traditional news media, trust in politics, voting intention and vote choice, social media use, traditional media use, and political orientation. Finally, they provided demographic information. (N = 989) | 1. Trust in traditional news media and trust in politics were negatively associated with belief in fake news.  2. Belief in fake news is positively associated with vote switching from the main political party to right-wing populists. | DV  Perceptions of familiarity, perceptions of truthfulness  IV  Trust in traditional news media, trust in politics, voting intention, vote choice, social media use, traditional media use, political orientation | Source credibility |

Table 2

|  | Accuracy | Sharing | Emotions | Repeated exposure | Altruism | Identity | Confirmation bias | Source credibility | Social endorsement | Conspiracy thinking | Motivated reasoning |
| --- | --- | --- | --- | --- | --- | --- | --- | --- | --- | --- | --- |
| Altay et al. (2022) |  |  |  |  |  |  |  |  |  |  |  |
| Apuke et al. (2021) |  |  |  |  |  |  |  |  |  |  |  |
| Buchanan (2020) |  |  |  |  |  |  |  |  |  |  |  |
| De Coninck et al. (2021) |  |  |  |  |  |  |  |  |  |  |  |
| Hughes et al. (2022) |  |  |  |  |  |  |  |  |  |  |  |
| Islam et al. (2020) |  |  |  |  |  |  |  |  |  |  |  |
| Laato et al. (2020) |  |  |  |  |  |  |  |  |  |  |  |
| Lantian et al. (2021) |  |  |  |  |  |  |  |  |  |  |  |
| Lin, et al. (2022) |  |  |  |  |  |  |  |  |  |  |  |
| Martel et al. (2020) |  |  |  |  |  |  |  |  |  |  |  |
| Melki et al. (2021) |  |  |  |  |  |  |  |  |  |  |  |
| Miller et al. (2016) |  |  |  |  |  |  |  |  |  |  |  |
| Osmundsen et al. (2021) |  |  |  |  |  |  |  |  |  |  |  |
| Pennycook & Rand (2019) |  |  |  |  |  |  |  |  |  |  |  |
| Pennycook et al. (2018) |  |  |  |  |  |  |  |  |  |  |  |
| Pennycook et al. (2020) |  |  |  |  |  |  |  |  |  |  |  |
| Ross et al. (2021) |  |  |  |  |  |  |  |  |  |  |  |
| Sterrett et al. (2019) |  |  |  |  |  |  |  |  |  |  |  |
| Su (2021) |  |  |  |  |  |  |  |  |  |  |  |
| Swire et al. (2017) |  |  |  |  |  |  |  |  |  |  |  |
| Traberg & van der Linden (2022) |  |  |  |  |  |  |  |  |  |  |  |
| Xiao et al. (2021) |  |  |  |  |  |  |  |  |  |  |  |
| Zhou & Shen (2022) |  |  |  |  |  |  |  |  |  |  |  |
| Zimmermann & Kohring (2020) |  |  |  |  |  |  |  |  |  |  |  |
